# Simulation training significantly improves the performance of novice students achieving the standard transesophageal echocardiography views

**DOI:** 10.1186/s12909-025-06917-1

**Published:** 2025-03-07

**Authors:** Aleksander Siniarski, Paweł Piątek, Aleksandra Karcińska, Alicia del Carmen Yika, Kamil Szostek, Tomasz Dziwiński, Maciej Stąpór, Karolina Golińska-Grzybała, Adam Piórkowski, Andrzej Gackowski

**Affiliations:** 1 Department of Coronary Artery Disease and Heart Failure, Institute of Cardiology, Jagiellonian University Medical College, Krakow, Poland; 2 St. John Paul II Hospital, Krakow, Poland; 3 Department of Automatic Control and Robotics, AGH University of Krakow, Krakow, Poland; 4 Medical Simulation Technologies, Krakow, Poland; 5 Department of Interventional Cardiology, Institute of Cardiology, Jagiellonian University Medical College, Krakow, Poland; 6 Department of Biocybernetics and Biomedical Engineering, AGH University of Krakow, Krakow, Poland; 7 Noninvasive Cardiovascular Laboratory, St. John Paul II Hospital, Krakow, Poland

**Keywords:** Transesophageal, Echocardiography, Simulation, TEE, Medical education

## Abstract

**Background:**

Transesophageal echocardiography (TEE) is an important cardiovascular imaging modality, offering detailed images surpassing other techniques, including transthoracic echocardiography. TEE's efficacy relies on skilled physicians operating the probe. Adequate TEE training is often overlooked, leading to potential patient discomfort or harm during TEE examinations. TEE simulators address this gap, providing a safe environment for practitioners to enhance their skills.

**Aims:**

Our study aims to assess the progress of TEE novices, namely medical students following a specific learning protocol with a TEE simulator, hypothesizing significant improvements in the time and movements required for simulated TEE views after training.

**Methods:**

The study protocol encompassed the selection of predetermined simulated TEE projections (*n* = 16), in accordance with the American Society of Echocardiography guidelines, to facilitate a comprehensive examination involving essential TEE manipulations. Students were provided with unrestricted access to video lectures and subsequently underwent the initial assessment (Test 1). Following this, they had a period of one to four weeks for training on the simulator, with unlimited access to TEE simulation. Test 2 was then administered, and calculations were conducted based on the discrepancies between both tests. Analytical parameters comprised the precision of the TEE view, examination time, and the randomity of probe movement (RAR index). Subsequently, two independent experts rigorously compared the two tests and graded each student's performance change as improvement, no change or worsening.

**Results:**

Twenty-six Jagiellonian University Medical College students (median age: 22.5, IQR: 22–24) in the 2nd to 6th year and 50% females, with no prior experience of echocardiography or TEE simulator use participated in the study. Students familiarized themselves with online lectures before executing an identical TEE examination protocol. Median duration between Test 1 and Test 2 was 18 days (IQR: 12–28).

First, after TEE simulated training, students exhibited a higher frequency of successfully completing the TEE examination without major errors (grade: passed) (P = 0.039). Additionally, precision significantly increased at Test 2 (P < 0.001). The total duration of the simulated TEE examination was significantly shorter in Test 2 than in the initial test (P < 0.001). Furthermore, the RAR index for ante/retroflexion was substantially lower in Test 2, while probe rotation remained similar between tests. Overall, student progress was evident in the majority of cases, with only 2 students showing no improvement after 1–4 weeks of voluntary self-training with the TEE simulator, and 2 more demonstrating a neutral outcome.

**Conclusions:**

The use of a TEE simulator is valuable for learning how to perform proper and safe simulated TEE examinations. It is effective even for students without prior echocardiographic experience, improving precision, shortening examination time, and reducing unnecessary movements during simulated TEE. TEE simulation is an ideal learning tool for both students and cardiovascular clinicians to minimize mistakes in their practice.

**Supplementary Information:**

The online version contains supplementary material available at 10.1186/s12909-025-06917-1.

## Introduction

Transesophageal echocardiography (TEE) is one of the cardiovascular imaging techniques (CV) that has gained widespread use in many medical scenarios. It allows the acquisition of more detailed, high-resolution images compared to transthoracic echocardiography (TTE). This is possible due to a better acoustic window located in the esophagus, in a short distance from the heart, and high-frequency transducer. It can be used electively and in life-threatening conditions when rapid and correct diagnosis is needed. In addition, TEE is essential to guide modern cardiac interventions. In all of these scenarios, the quality of the TEE study is highly dependent on the skill of the physician who operates the probe and interprets the images. Therefore, future cardiologists and anesthesiologists need adequate TEE training.

The probe has five degrees of freedom: 1). depth of the probe, 2). rotation in the esophagus, 3). anterior vs. posterior tip flexion, 4). left vs. right tip flexion ultrasound plane angle rotation (0–180 degrees range). All must be coordinated to obtain a proper view. However, the position and orientation of the probe tip are not visible during the exam and the manipulations are based on the ultrasound image only. The esophagus trajectory is curved, and the heart axis is oblique in the thorax. All of the above make learning the TEE difficult [1, 2].

TEE training is often neglected since it requires hours of practice manipulating the probe, experienced teachers, and is usually not feasible in the setting of a limited time during the procedure. If an examination is performed by a physician without proper training and experience, it may cause unnecessary patient discomfort or even harm, which may result in serious complications, such as esophageal perforation and mediastinal infection. While TEE is generally considered a semi-invasive, but relatively safe examination, complication rates can vary depending on the physician's experience. A prolonged manipulation of the TEE probe is an independent contributing factor to major TEE complications, including hemodynamic compromise and upper gastrointestinal bleeding requiring transfusions [3]. TEE simulators respond to the needs of ultrasonographers and allow them to improve their skills in a safe environment. According to the results of some studies, training on those simulators allowed students and medical residents to achieve projections more efficiently and confidently [4, 5]. In addition, practical TEE training improved the interpretation of images and pathologies that were assessed by the tests [6, 7]. Previous studies primarily compared people who had undergone TEE simulator training with those who did not.

The purpose of our study was to determine the progress that individuals new to the TEE exam made after following a specified learning protocol and to compare the results before and after training. Our hypothesis is that after training, the time and number of movements required to obtain TEE views will decrease significantly, reflecting the improvement in TEE skills.

## Methods

### Study samples

The study included 26 medical students from the Jagiellonian University Medical College in Krakow, Poland, who had basic knowledge of heart anatomy but had never been trained in echocardiography. They voluntarily wanted to learn the TEE principles and test themselves on the MrTEEmothy® TEE simulator twice: a) after the basic TEE lecture but before manual TEE training, and b) after a customized automated training, based on the American Society of Echocardiography (ASE) recommended protocol for TEE views.

### Study protocol

For each specified projection, the positioning of the imaging plane was delineated by a proficient echocardiographer (with > 15 years of experience in TEE) through the placement of 3 distinct points defining the plane. In principle, the optimal view necessitates the inclusion of all three points. To establish an acceptable tolerance for the plane positioned by the student during the exercise, the limit of distance between each defined point and the tested plane was predetermined before the study. This determination was executed by two independent experts (both > 15 years of experience in TEE). One expert ascertained the individual distances from the points to the plane, ensuring that if the plane fell within the prescribed distance limits, the anatomical landmarks crucial for each view would be adequately visible on the sector. The second independent expert systematically assessed all views to verify their appropriateness, and both experts adjusted the limits through consensus. The precision of plane positioning was electronically quantified throughout the exercise and documented in the study logs. An 80% cutoff limit was deemed adequate for accepting the given view. All probe movements (including depth, probe rotation, ante-retro flexion, right-left flexion, and plane seek angle) were continuously recorded in digital format by simulator software, enabling retrospective review and analysis.

To streamline the protocol for novice students, a subset of 16 views was chosen from the comprehensive set of 28 views recommended by the ASE guidelines [1, 2]. The selection process was arbitrary, with a focus on incorporating views that, while relatively easy to acquire, are clinically very important in most of the TEE studies. The set of views requires positioning the probe at three different levels of esophagus, various rotations of the probe in the esophagus, and the selection of the omniplane angle and optimal ante-retro-flexion of the probe. The sequential arrangement of these selected views within the protocol was concordant with the ASE guidelines [1, 2] and included: 1) Mid-esophageal (ME) 5-chamber, 2) ME 4-chamber, 3) ME Mitral Commissural, 4) ME Mitral 2-chamber, 5) ME mitral long-axis (LAX), 6) ME aortic valve (AV) LAX, 7) ME ascending (Asc) aorta (Ao) LAX, 8) ME Asc Ao short axis (Asc Ao SAX), 9) Right ventricle (RV) inflow-outflow view; 10) ME AV SAX, 11) ME Bicaval, 12) Transgastric (TG) Basal SAX mitral valve, 13) TG mid-papillary SAX, 14) TG 2ch, 15) Desc Ao SAX, 16) Desc Ao LAX. The sequence allows ergonomic transition from one view to the next one in a group, with limited motions in the esophagus. Such modifications are easier to learn compared to obtaining the view in a different group. This is why, to assess more difficult aspects of the training, the 4 most relevant views from 4 different groups were selected for additional analysis. They included: 1). ME mitral commissural, 2). ME bicaval, 3). ME asc Ao, 4). TG basal SAX views. To pass the test, final precision > 80% (with the optimal view held for at least 3 s) was required in all 16 TEE views.

Preceding the exercise, a TEE expert delivered a didactic lecture to all participants through a recorded video format (link: https://www.youtube.com/watch?v=BNXlcPXLZog&list=PL1eBZ4rl7LHnFGZJ2rmUOujdA68W_LdEb). Each student was afforded unrestricted access to this instructional video in advance of the assessment (prior to both tests 1 and 2). Subsequently, each student underwent initial 45 min training to acquaint themselves with the movements of the TEE probe and familiarize themselves with the operation of the TEE simulator. The simulator provided automated guidance on the requisite probe movements to obtain specific views in a predetermined sequence.

After initial instruction, students underwent an first proficiency test (test 1) to establish their baseline performance, documented for evaluation. Subsequently, they were granted access to the simulator for individual, unrestricted practice sessions over the course of one to four weeks. After this practice period, each student underwent a second proficiency test (test 2) to assess any improvement in their skills. To maintain anonymity, each student was assigned a unique anonymized nickname and had no access to their previous test results.

Simultaneously, the expert cardiologist conducted a similar proficiency test, serving as the gold standard reference. The results of each student's tests were then compared against the expert's performance, and changes in the students' proficiency were systematically analyzed.

### Transesophageal echocardiography simulator

During this project, MrTEEmothy® (Medical Simulation Technologies, Krakow, Poland) was employed, incorporating tailored presets designed to align with the specific requirements of the research project. Within the TEE simulator, the "EduTool" was deployed, employing well-established echocardiographic projections that adhere to the prevailing guidelines set by the ASE [1].

### Methods of obtaining raw data from the simulator

**Fig. 1:**
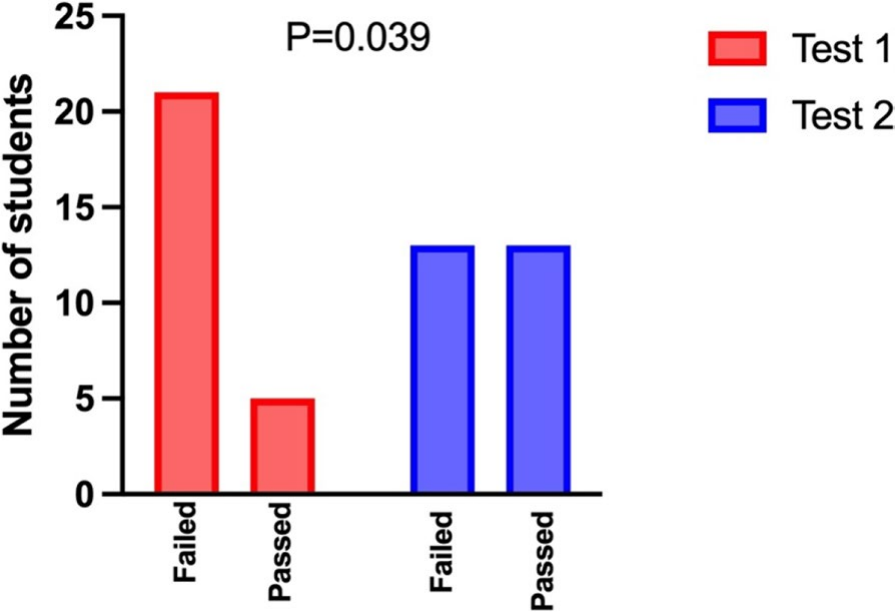
The number of students who failed and passed the TEE examination before and after the simulation training. The data is presented as raw numerical values indicating students who either failed or passed the entire transesophageal echocardiography (TEE) examination protocol. Students undergoing Test 1 are denoted in red, while those undergoing Test 2 are distinguished in green. To pass the test, final precision > 80% (with the optimal view held for at least 3 s) was required in all 16 TEE views

The MrTEEmothy simulator comprises a phantom, a laptop, and a TEE probe that is a generic version of real TEE probes operated in examination (Fig. 1). Users interact with the probe in a similar way to real-life scenarios: The probe can be advanced or withdrawn from the phantom’s esophagus to change the depth of the transducer. Coaxial knobs control ante- and retroflex and left–right bend, while two buttons on one side of the handle change the plane angle. The rotation of the probe handle is measured with precise sensors.

All user probe manipulations are recognized by the software installed on the laptop and used to calculate the position and rotation of the virtual transducer. Then the simulation is performed and an ultrasound projection is rendered accordingly. During training, separate time series are logged from every probe sensor: including probe rotation, plane angle, depth, and both bends (anteflexion/retroflexion and right/left). Furthermore, during the data recording process, more information is stored in logs, including a 'precision factor': the time needed to obtain the given view.

### Methods of complete and individual data analysis

One of the parameters for assessing this discomfort is the TEE diagnostic time. Another important factor increasing the patient's discomfort and oesophageal injury risk is the rapidity and randomity of movement of the TEE probe. Due to the design, the motion of the probe can be described in two coordinate systems. In the first case, the position of the TEE probe can be described by the following coordinates:

- probe insertion depth (Probe Depth)
- probe rotation angle (Probe Rotation),
- angle of bending the front-back probe (Anteflex—Retroflex),
- probe bending angle left–right (Probe Left—Right).

In the second coordinate system, the position of the probe can be described in the Cartesian coordinate system by the coordinates: x, y, z, which describe the position of the probe in three-dimensional space.

### Average precision analysis

The average (mean or median) precision for a specific projection was calculated as the average of all precision signal samples for that projection (obtained every 100 ms). The average precision for a test was calculated as the average of the mean precision values for each projection in the respective test (Supplementary Fig. 1).

### Rapidity and randomity movement (RAR Index)

To assess the rapidity and randomity of the movement, the first coordinate system was used. The coordinates selected for analysis were:

- probe depth,
- probe rotation,
- probe bends

Changing the remaining coordinate (ultrasound plane rotation) does not affect the discomfort of the TEE diagnostic or this effect is negligible.

The cumulative absolute value of the differential of the individual coordinates was used as a measure of the rapidity and randomity of the probe movement (RAR index). This index can be written as:

$$RAR=\sum\nolimits_{n=0}^N\left|\frac{{ds}_n}{dt}\right|,$$

Where the value of the finite difference is expressed by the formula for the central difference:

$$\frac{ds}{dt}=\frac{{s}_{n+1}-{s}_{n-1}}{{t}_{n+1}-{t}_{n-1}}$$

Where:

$s$- signal of the analyzed coordinate,

$$t$$- time signal,

$N$- number of signal samples of the analyzed coordinate,

According to the described formula, the value of the RAR index is an increasing function and its value depends on the sum of the changes in the coordinate analyzed over a time period. The RAR index was calculated for the three coordinates previously described defining the position of the TEE probe. The RAR index can be analyzed as a numerical value for the time in which a projection is achieved and as a function represented in the time domain.

### Qualitative analysis of the improvement/worsening (global TEE examination assessment)

Finally, a consolidated examination of all student outcomes was executed to discern individuals exhibiting superior aptitude relative to those demonstrating diminished TEE proficiency. Furthermore, enhancements between the initial and subsequent assessments were appraised for each participant.

For each view in both tests, a comprehensive analysis of precision, RAR index, and the time required to obtain a view was conducted through blind evaluation. The comparison was quantified as worsening (−2 or −1), no change (0), or improvement (+ 1, + 2). In instances of discordance between the experts, a re-analysis was undertaken, and the final grading was resolved through consensus.

Our arbitrary, global, qualitative measurement of TEE examination was graded as follows: −2 points if significant deterioration, i.e. time more than twice as long, precision less than 80%, RAR index greater than 30%; −1 points if deterioration, i.e. time longer by > 5% compared to the initial one or deterioration of precision < 80% or jerking twice as bad; 0 points if without significant change, i.e. time difference ± 5% of the output time and maintained accuracy > 80% and RAR index ± 30%; + 1 points if improvement, i.e. the time of Test 2 is shorter by 5–99% compared to the initial Test 1 and the quality of the test assessed by precision (> 80%) and low RAR index are maintained; and + 2 points if significant improvement, i.e. time 2 × shorter compared to Test and accuracy > 80%, RAR index not worse than in Test 1.

### Statistical analysis

Continuous variables were presented as mean ± standard deviation (SD) or median (interquartile range, IQR), while categorical variables were expressed as numbers (%). The normality of distributions was assessed using Shapiro–Wilk tests. Inter-group differences for continuous variables were determined using Student's t-test or Mann–Whitney U test. First, the calculations were performed for all views attempted by a given student in both tests and compared. Then, a detailed analysis was performed for selected, most representative 4 TEE views: 1). ME mitral commissural, 2). ME bicaval, 3). ME asc Ao, 4). TG basal SAX views. Statistical evaluation was performed in IBM SPSS version 29.0.0.0 (Armonk, NY, USA) and GraphPad Prism version 10.1.1 (San Diego, CA, USA), licensed for A.S.

The sample size was calculated based on the previously published study [4]. The primary endpoint of that study was the change in total assessment scores in TEE simulation between analysed groups. Choosing a power of 80% and a 2-sided alpha level of 0.05, at least 15 patients in each group were required.

## Results

Twenty-six students from the Jagiellonian University Medical College aged 22.5 years (IQR: 22- 24), with 50% of females (*n* = 13), during the 2nd to 6th year of medical studies (the majority during 4th year) in a 6-year program at the Faculty of Medicine, actively participated in the study. Students attested to having no prior exposure to related coursework, practical experience in echocardiography, or utilization of any TEE simulator. In adherence to the study protocol, students were afforded ample time for familiarization with initial instructional materials, consisting of a series of online lectures elucidating the principles of TEE testing and the simulator's operation. All students executed the identical TEE examination protocol. The median duration between Test 1 and Test 2 was 18 days (IQR: 12–28 days).

### Precision

The aim of the exercise was at least 80% concordance of the obtained view plane with the “ideal” view, otherwise the attempt was considered as “failed”. The number of views with success or failure was calculated in both tests. The number of successful attempts increased significantly at Test 2 compared to test 1 (Fig. 1).

**Fig. 2:**
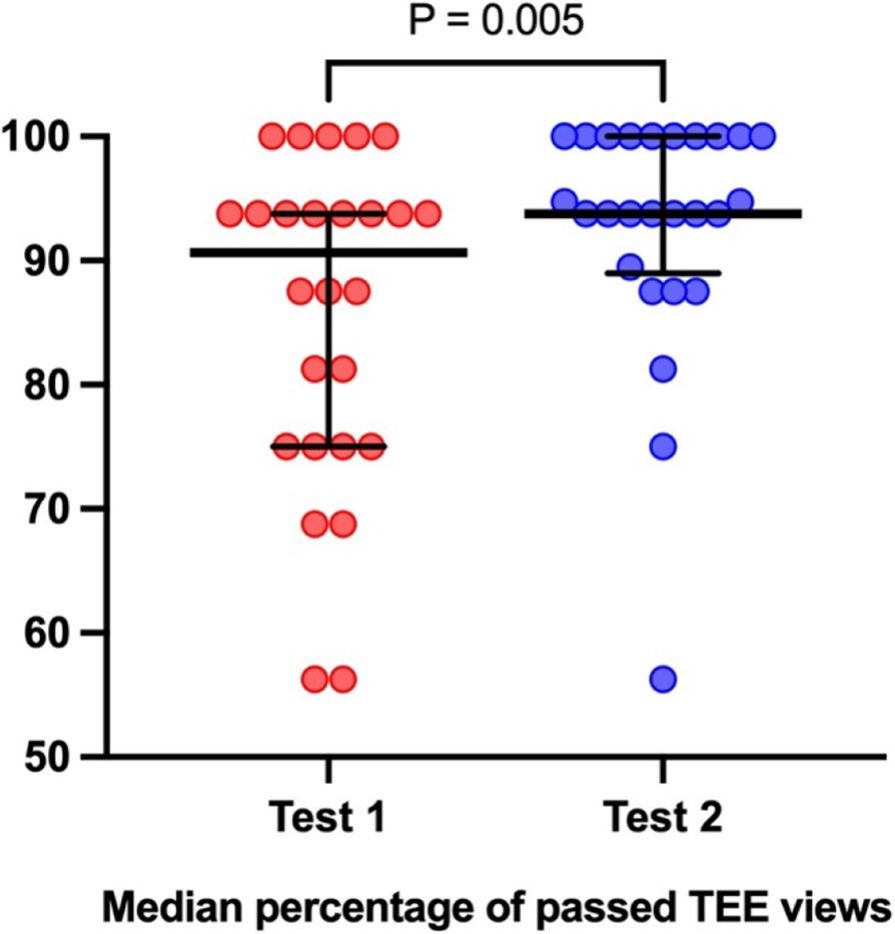
Comparison of the median percentage of precision values from Test 1 and Test 2. The data is presented as the percentage of successfully passed transesophageal echocardiography (TEE) views within the entire examination protocol. A TEE view was considered passed when the final precision of the analyzed view equaled or exceeded 80%. The number of these successfully passed TEE views was divided by the maximum number of performed views (n = 16) to express them as a percentage. The vertical bars denote the median values, and the whiskers represent the interquartile range. Red dots correspond to all students’ attempts in Test 1, while blue dots represent students' attempts in Test 2

Moreover, we assessed the median percentage of successfully achieved TEE views (with a maximum value of 16), excluding cases involving esophageal perforations, wherein the examination view was deemed unsuccessful. Subsequently, we compared the outcomes of test successes between Test 1 and Test 2. A noteworthy enhancement was observed, with documented success rates of 90.6% and 94% in Test 1 and Test 2, respectively (Fig. 2).

**Fig. 3:**
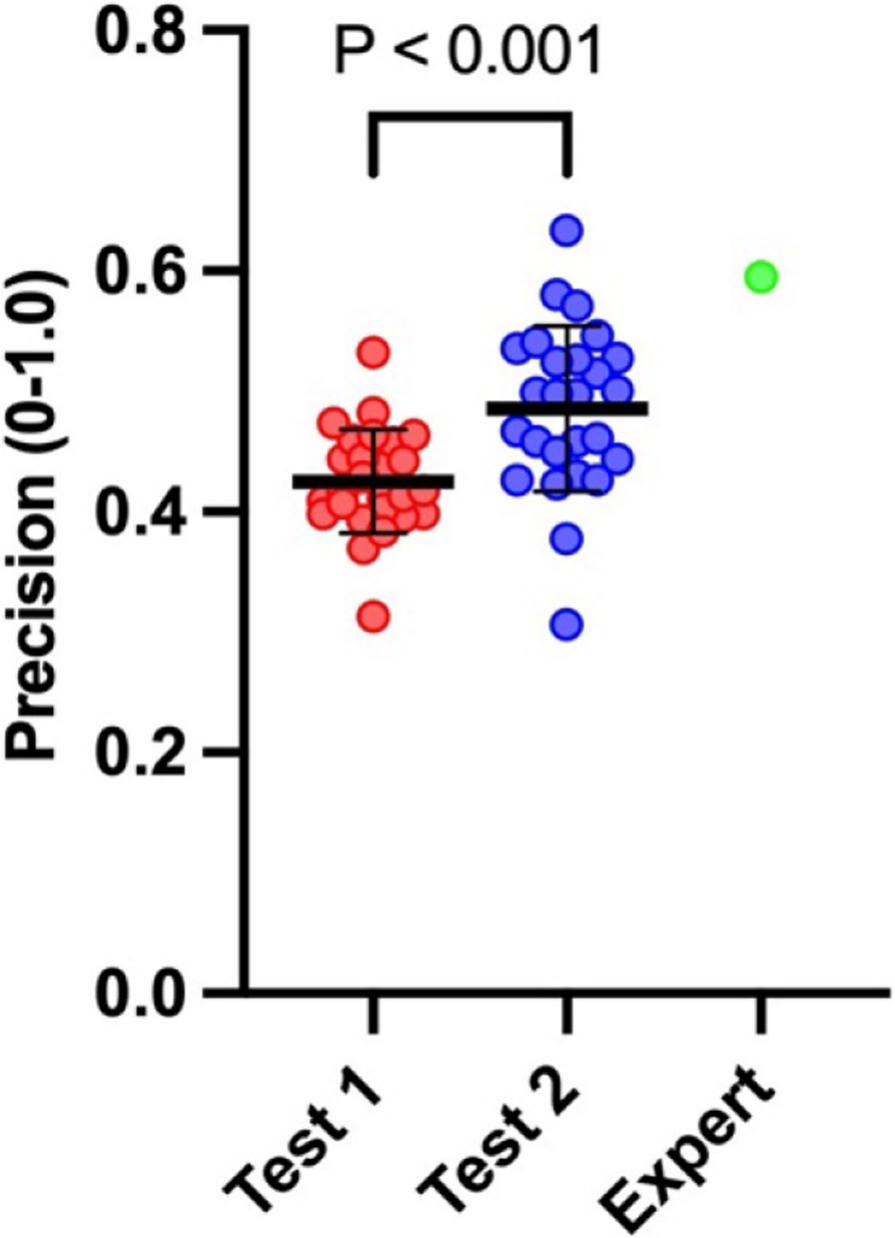
The mean values of precision throughout the entire simulated TEE examination for both tests and the expert. The data is depicted using means (bold horizontal bars) and standard deviations (whiskers). The figure illustrates mean precision values (derived from each student from each second of every TEE view) for the entire simulated TEE examination for each student and expert. Red dots correspond to all attempts by students in Test 1, whereas blue dots represent attempts in Test 2

The sum of precision measurements for all views obtained by each student was analyzed and subsequently the mean values were compared between both tests. The analysis revealed a statistically significant increase in the total precision for the entire simulated TEE protocol (0.43 ± 0.043 vs. 0.48 ± 0.07, Fig. 3).

**Fig. 4:**
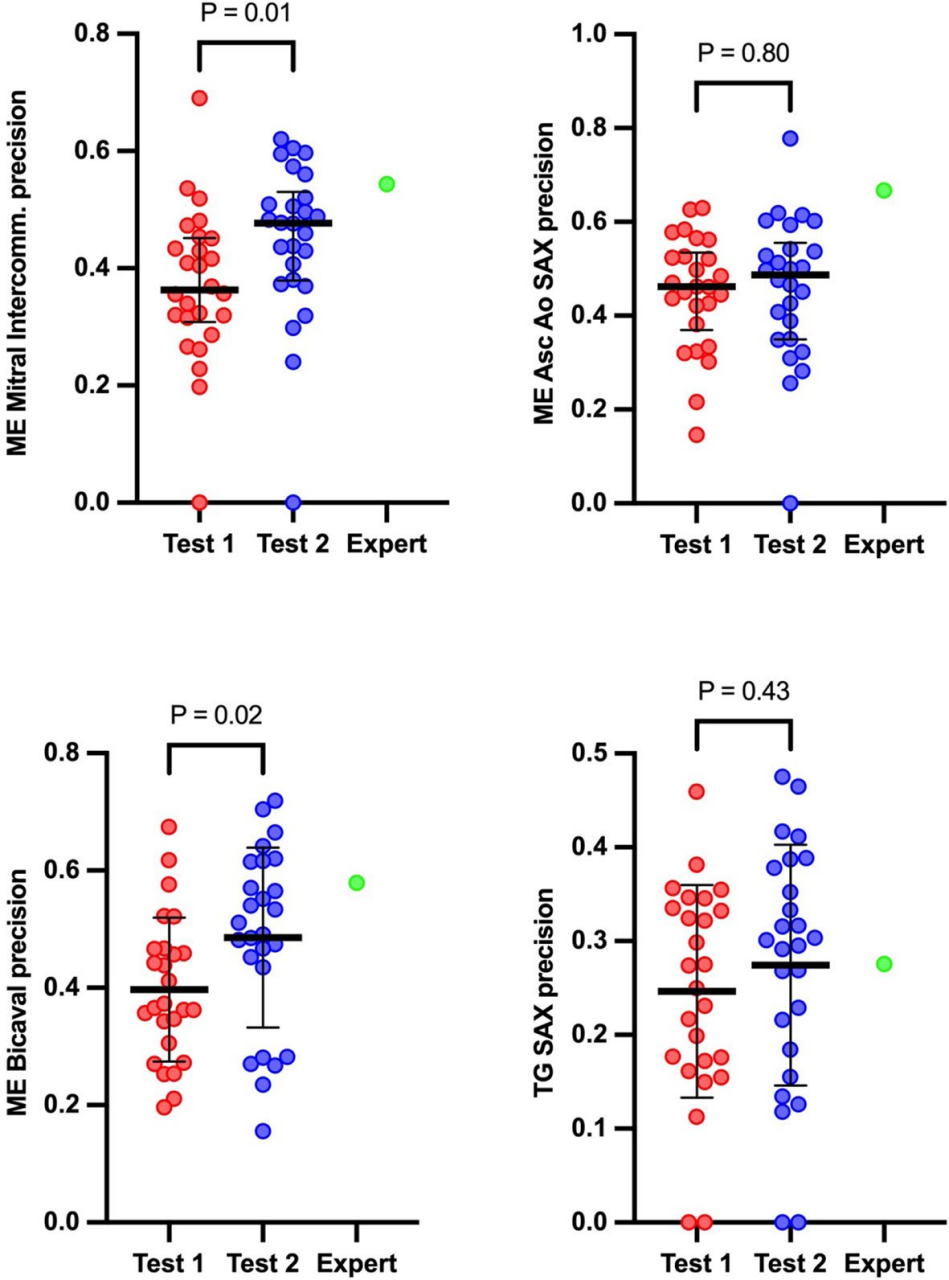
Comparison of the precision in four selected TEE views for both tests and the expert. The data is represented using means or medians (shown as bold horizontal bars) and standard deviations or interquartile ranges respectively (indicated as whiskers). The figure illustrates the median or mean precision values derived from each student for each second of analyzed TEE view. The red dots correspond to all attempts by the students in Test 1, while the blue dots represent the attempts in Test 2

A detailed analysis of selected four single TEE projections, obtained at different levels of esophagus, was conducted for each student during Test 1 and Test 2. A notable improvement was identified in two specific TEE views, namely the ME Mitral Commissural (from 0.36 [IQR: 0.31–0.45] to 0.48 [IQR: 0.38–0.53]) and ME Bicaval (from 0.40 ± 0.12 to 0.49 ± 0.15) (Fig. 4).

### Time needed to obtain the views

**Fig. 5:**
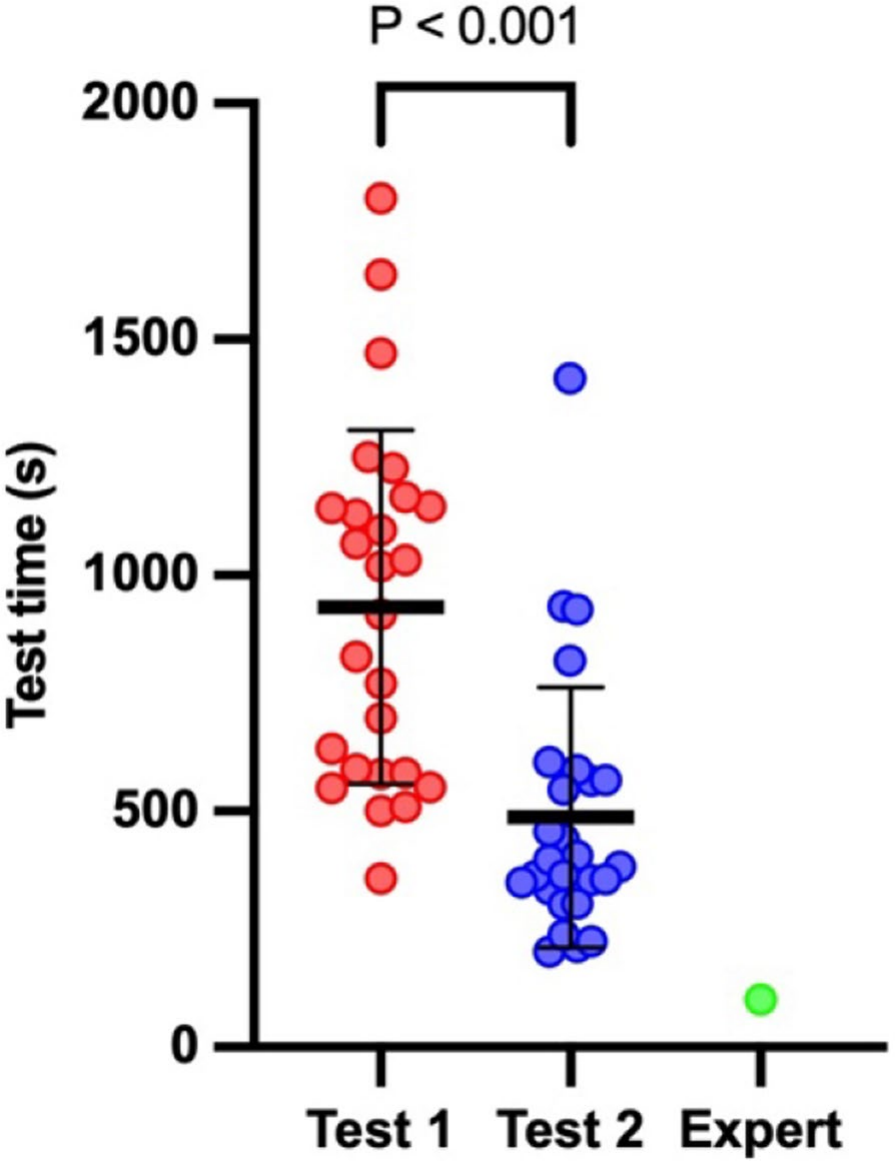
Total time to complete the TEE examination between the tests and an expert. Data are depicted using medians (bold horizontal bars) and interquartile ranges (whiskers). The figure illustrates the total time values (s) for the complete TEE examination. The red dots correspond to all attempts by the students in Test 1, while the blue dots represent the attempts in Test 2; the expert is marked as a green dot

The total time needed to obtain all views in a sequence by a given student was calculated. The comparison showed a highly significant decrease in the time needed to obtain a complete (as per protocol) TEE examination between Test 1 and Test 2 respectively (968 s (IQR: 580–1151 s), 390 s (IQR: 327–571 s), Fig. 5). The students’ results are also compared graphically with an expert (> 15 years of experience in TEE).

**Fig. 6:**
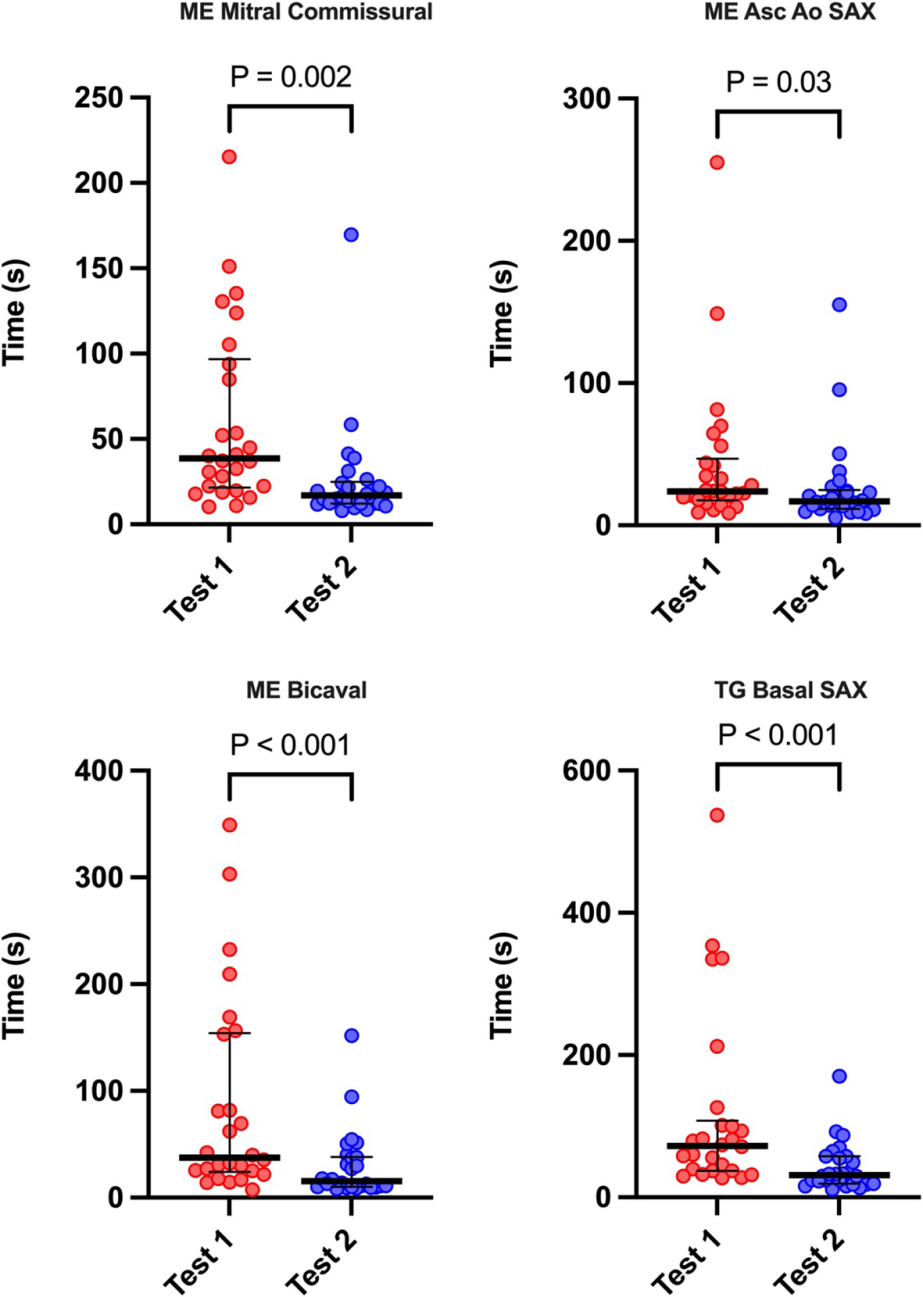
Time of completion of selected TEE views in both tests. Data are depicted using means or medians (bold horizontal bars) and standard deviations or interquartile ranges (whiskers). The figure illustrates the time needed to complete chosen TEE view (s). The red dots correspond to all attempts by the students in Test 1, while the blue dots represent the attempts in Test 2

We have also analyzed four selected TEE views to demonstrate the difference in these representative projections. We showed a systematic and significant improvement in the measured time for the four TEE views (Fig. 6). The values for each view, respectively for Test 1 and Test 2, were as follows: for ME Mitral Commissural view: 39 s (IQR:22–97 s) and 17 s (IQR: 12–25 s); ME Asc Ao SAX view 24 s (IQR: 18–47) and 17 s (IQR: 12–25); ME Bicaval view 38 s (IQR: 24–154 s) and 15 s (IQR: 10–38 s); and for TG Basal view 72 s (IQR: 37–108 s) and 31 s (IQR: 19–6 s) (Fig. 6).

### Rapidity and randomity of the probe movement (RAR index)

**Fig. 7:**
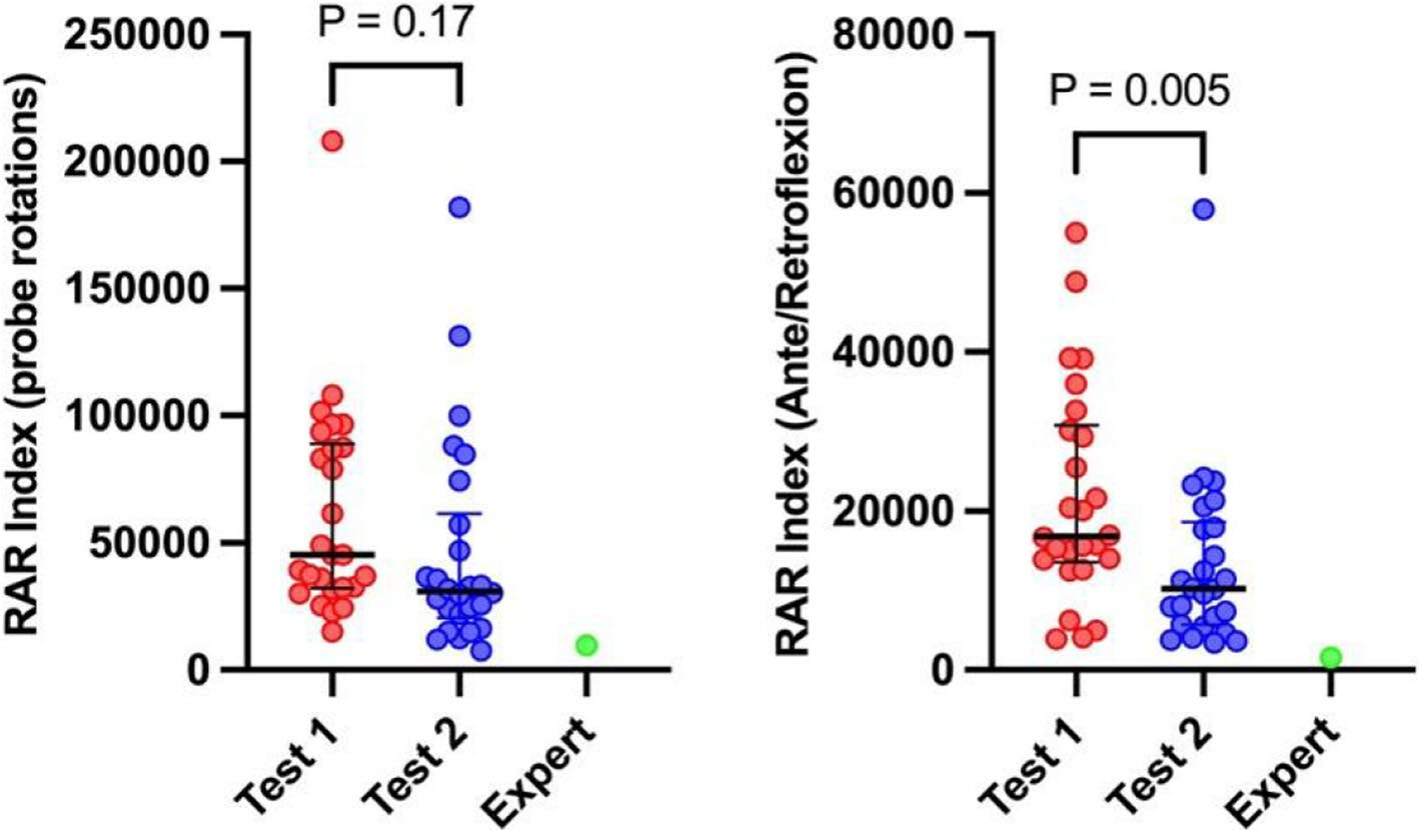
The rapidity and randomity of the probe movement indexes for probe rotation and ante-retroflexion for a complete TEE examination. Data are depicted using medians (bold horizontal bars) and interquartile ranges (whiskers). The figure illustrates the RAR index for complete TEE examination from each student. The red dots correspond to all attempts by the students in Test 1, while the blue dots represent the attempts in Test 2; the expert is demonstrated as a green dot

The RAR index was calculated for each attempted probe motion across different views. The cumulative sum of RAR index values for a specific probe motion throughout the entire test for each student was analyzed and subsequently compared between Test 1 and Test 2. A trend toward improvement, although without statistical significance, was observed for probe rotation. In contrast, a statistically significant improvement was noted for ante-retroflexion of the probe (Fig. 7).

**Fig. 8:**
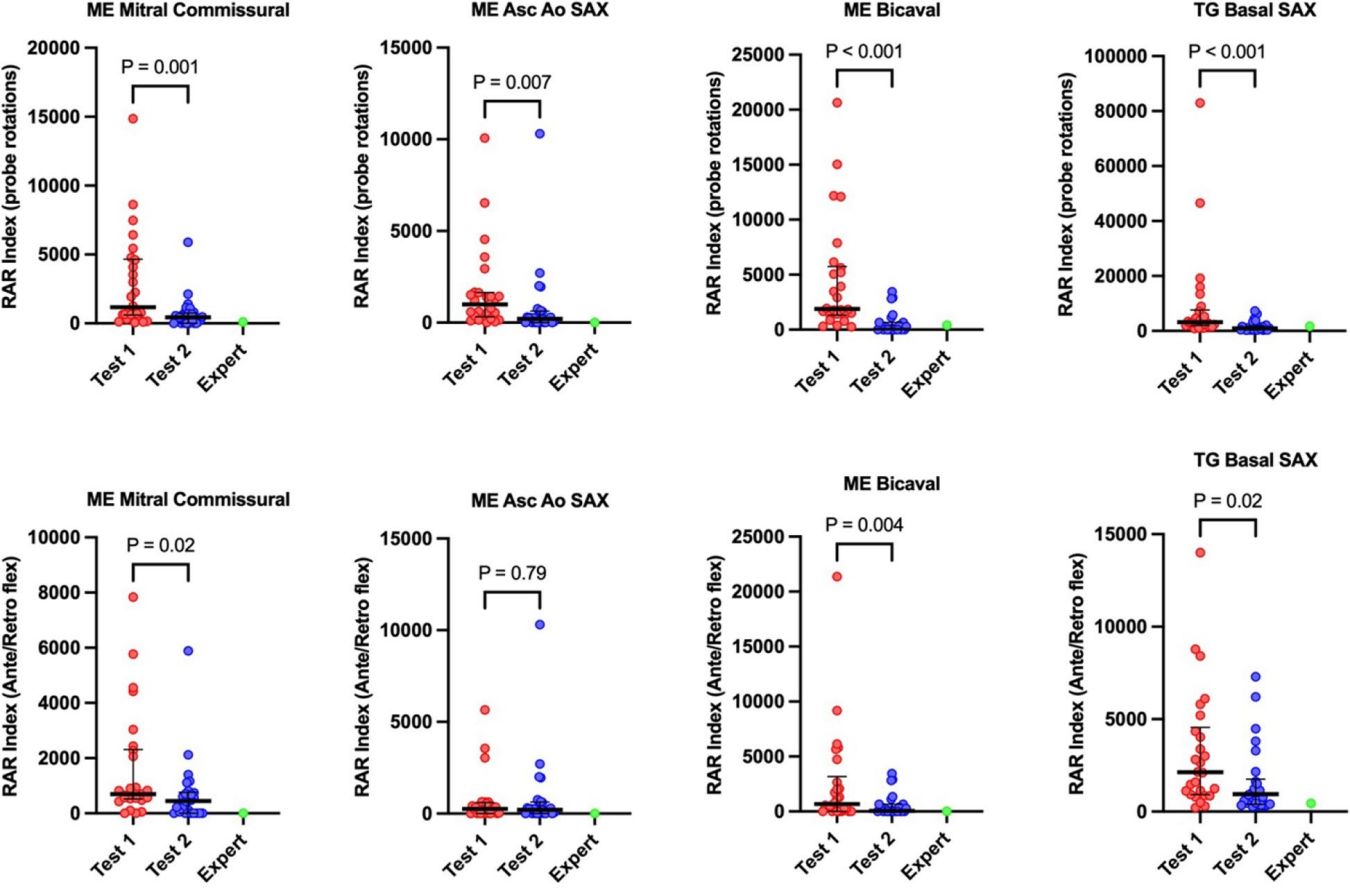
Rapidity and randomity index of the probe movement indexes for probe rotation and ante-retroflexion for selected TEE views. Data are depicted using medians (bold horizontal bars) and interquartile ranges (whiskers). The figure illustrates the RAR index for probe rotation (top) and ante-retro flexion (bottom) for selected four TEE views. The red dots correspond to all attempts by the students in Test 1, while the blue dots represent the attempts in Test 2; the expert is demonstrated as a green dot

Notably, in a subanalysis of four selected TEE views, we demonstrated a significant decrease in the RAR indexes for probe rotation for all projections between Test 1 and Test 2 (Fig. 8). The specific RAR indexes for probe rotation in selected four TEE views, respectively for Test 1 and Test 2, were as follows: for the ME Mitral Commissural view: 1177 (IQR: 601–4652) and 525.5 (IQR: 250.8–1972); ME Asc Ao SAX view 990.5 (IQR: 314–1640) and 697 (IQR: 288.5–1217); ME Bicaval view 1892 (IQR: 1313–5749) and 1586 (694–3591); and for the TG Basal SAX view 3225 (IQR: 2066–7604) and 2152 (IQR: 1412–4816) (Fig. 8, top). A similar analysis was performed for the ante-retroflexion RAR index. Specific RAR indexes for ante-retroflexion of the probe in selected four TEE views, respectively for Test 1 and Test 2, were as follows: for the ME Mitral Commissural view: 695 (IQR: 518–2313) and 445 (IQR: 0–761); ME Asc Ao SAX view 260 (IQR: 0–600) and 210 (IQR: 0–630); ME Bicaval view 680 (IQR: 0–3173) and 60 (0–653); and for the TG Basal SAX view 2125 (IQR: 913–4558) and 940 (IQR: 390–1760) (Fig. 8, bottom).

### Qualitative analysis of the improvement/worsening

**Fig. 9:**
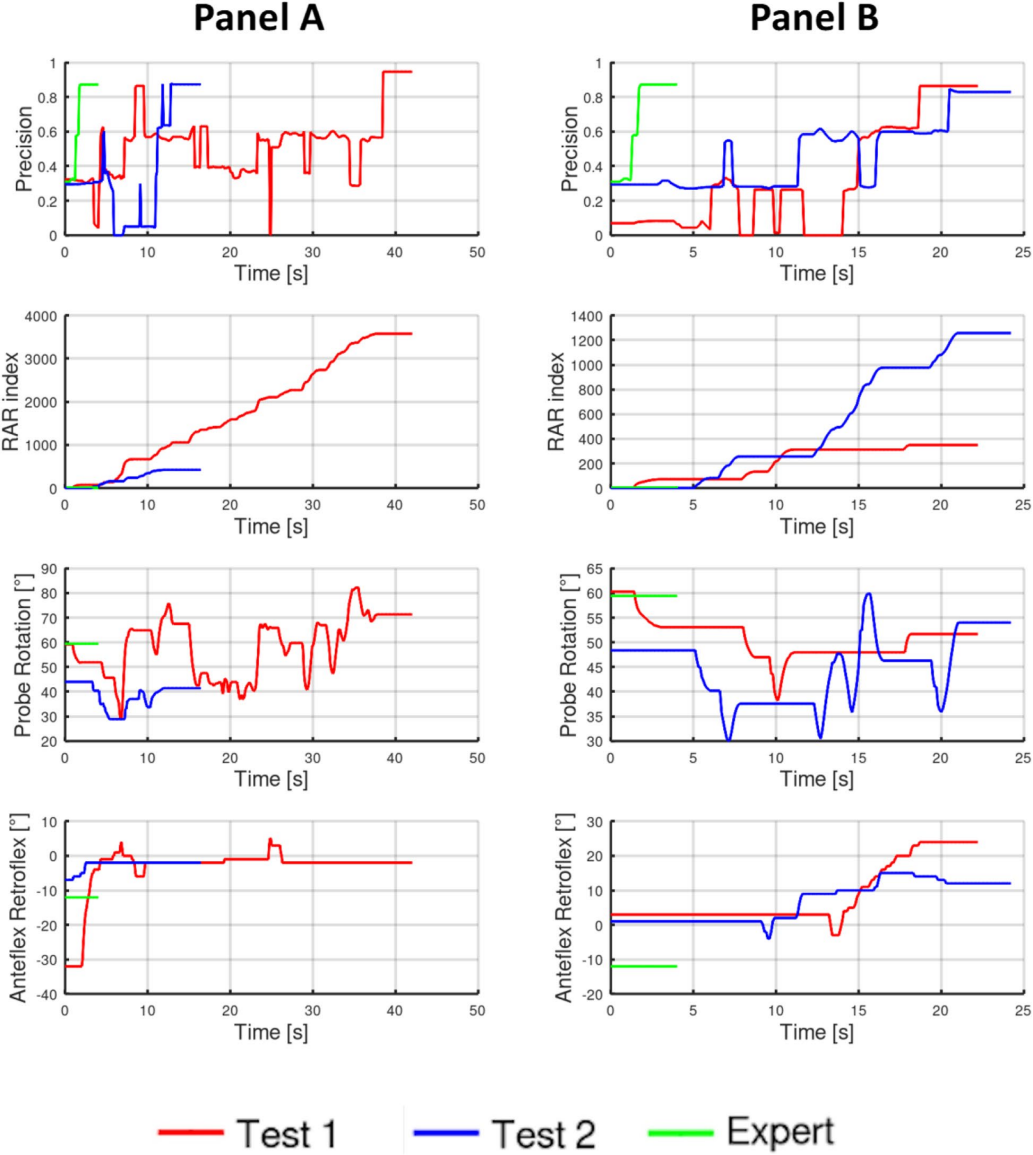
Examples of individual student’s graphs of selected TEE view before and after the TEE training compared to the expert. **A** example graphs of the parameters for a given view, analyzed by 2 experts in order to qualify the student’s performance as a +2 improvement. Red lines represent test 1 probe motions, dark-blue – test 2, in comparison to the expert (green line). The time to achieve the view shortened more than twice; there were significantly less maneuvers and the RAR index was lower for test 2 compared to test 1, however the expert’s metrics were even lower. **B** example graphs of the parameters for a given view, analyzed by 2 experts in order to qualify the student’s performance as a −2 worsening. Red lines represent test 1 probe motions, dark-blue – test 2, in comparison to the expert (green line). The time needed to achieve the view was twice longer in test 2, and the RAR index was higher for test 2 compared to test 1

All 26 students were individually assessed based on a graphs from each TEE view by two independent experts. The analysis of charts, considering the completion of projections, examination time, precision, and RAR index, formed the basis for issuing the final evaluation. It was demonstrated that the average improvement in the performance of the assumed simulated TEE examination was + 0.96 (SD = 0.73). Based on pre-established criteria, it was determined that 22 students enhanced the quality and speed of the TEE examination, 2 remained neutral, and 2 exhibited a decline in performance (Fig. 9).

## Discussion

The results of this original research have shown that students with no previous echocardiographic training can be effectively trained to obtain basic and more complicated TEE views. Not only the precision of the TEE views, but also time needed to obtain complete TEE examination (16 prespecified views), as well as RAR indexes, which is a surrogate marker of possible complications during TEE examination, improved significantly after only one to four weeks of simulated training. To the best of our knowledge, this is the first study comprehensively assessing the advancement of medical students who were previously unfamiliar with any echocardiographic courses and any TEE training. Utilizing the capabilities of the MrTEEmothy® TEE simulator, we were able to meticulously demonstrate and analyze every second of each view throughout the entire TEE examination.

These findings align with previous studies demonstrating the effectiveness of simulators in enhancing both practical and theoretical skills. Technological advancements now offer secure training environments for inexperienced doctors or students, improving outcomes during invasive procedures. While medical simulators are commonly used in universities, many lack validity assessments. Our goal was to evaluate the efficacy of the TEE simulator presented here for medical education, assessing its potential for continued use in cardiology [8].

Other studies have shown that e.g.: surgical simulator training reduces procedure time in real life compared with conventional training [9, 10]. Semi-invasive procedures such as TEE demand thorough training. Simulators enable echocardiography skills practice without constant oversight, addressing challenges like limited access to experienced teachers. While mentor support remains integral, automated simulated protocols can efficiently replace fundamental training elements.

Importantly, enhancing technical skills prior to real patient studies is the most important benefit of learning through any simulation. In terms of TEE, simulated training mitigates patient discomfort and reduces the risk of esophageal injury from unnecessary probe motions. Neelankavil et al. demonstrated improved image quality and better identification of anatomic structures and correct views in medical residents after TEE simulation [11].

TEE is crucial for diagnostic and monitoring purposes, demanding extensive training for proficient probe manipulation and accurate diagnostics. Essential for structural cardiac interventions, such as transcatheter mitral/tricuspid repair or left atrial appendage closure, practical skills are vital due to the high image quality and the clarity of anatomical structures [12–14]. In the study performed by Prat et al., the acquisition of manual skills by the intensive care unit (ICU) trainees was accelerated after the introduction of a simulator-based training program [15]. Sohmer et al., compared pre-training TEE simulator and written examination scores between the self-guided and instructor-guided groups, the post-training performances between those groups as well as pre- vs post-training [16]. Although the median pre-training results were similar, the median post-training score was significantly improved for both. Moreover, no significant improvement difference was noted between self and instructor-guided groups. There is no doubt that training with a TEE simulator enhanced the quality of the performed procedure, being superior to other learning tools used in the study. The pre-test clearly showed a lack of necessary manual and psychomotor skills. Damp et al., performed a study in which groups with simulator training had higher total assessment scores than group with standard TEE training [4]. Moreover in the study performed by Ferrero et al., showed that medical residents after lecture based or simulator training obtained 10 commonly used standard views on an anesthetized patient [17]. The quality of the images was remarkably higher in the simulator group than in the control group [17]. After training, TEE knowledge was also significantly higher, but with no difference between the simulation group and the group who received training during cardiac surgery [18].

Silva Restrapo et al. reported improved post-training outcomes among trainees after a one-day educational course on a critical care TEE simulator [19]. The study assessed image acquisition quality, but not the time required for acquisition. Given the need for proficient performance in semi-invasive procedures, intraoperative TEE monitoring, and critical cardiac care interventions, our study aimed to determine if simulator training significantly reduces the time required for obtaining views. The results showed a substantial reduction in time, lowering the risk of complications associated with prolonged TEE examinations.

While the majority of students in our study demonstrated significant improvement, some did not, emphasizing the importance of individual performance assessments for objective skill evaluation. This suggests a potential need for additional, modified, or extended training. Ensuring proper skill development is critical not only for individual career planning but also to prevent inadequate patient examinations. Separate studies should investigate this aspect further. Although our study highlights improved image acquisition and reduced TEE view acquisition time with the simulator, the clinical utility remains unknown and warrants future exploration. Identifying the most efficient training schemes, considering the minimum use of simulator time and intervals between sessions, is crucial for consistent outcomes. While the presence of an experienced tutor may enhance training, it can complicate scheduling. Some studies report no significant impact based on the instructor's presence or absence. Allowing simulator use at any time could alleviate availability and time constraints. Ongoing discussions should determine the optimal integration of TEE simulations into medical residency programs and which specialties benefit most. In conclusion, medical simulators are vital for education, especially in TEE training, but further research is needed to optimize programs for medical students and residents.

### Limitations

Our study has some limitations. First of all, this was a single-center study experience and our sample size was relatively small. Therefore, no additional subgroup analyses are statistically justified and were not performed. It is important to take into account several factors, such as motivation, commitment, and peer support, which undoubtedly had an impact on the results. It is also possible that the outcome is influenced by the time difference between the stages of the protocol in a particular individual (e.g.: a few days vs 2-week difference).

Pre vs post-test comparisons of the quality of obtained images, the level of understanding, proper image interpretations, satisfaction, skills improvement, and pathology recognition weren’t assessed. 14 key-projections were used (the most advanced were excluded). Additionally, only results obtained after training on the specific simulator (MrTEEmothy® by Medical Simulation Technologies, Krakow, Poland) were compared, thus the outcomes can vary depending on the simulator type/brand, including presets, when compared to real-life TEE examination. Finally, despite its undoubted enhanced technological benefits, the TEE simulator used remains an artificial device, which raises questions about how skills learned could be transferred to real-life situations.

## Conclusions

We have demonstrated that short practical training sessions on the TEE simulator led to a significant reduction in the time required to complete the TEE examination, along with increased precision and reduced random, unnecessary, manipulations of the TEE probe within the esophagus. A detailed analysis of students' performance revealed that the vast majority of individuals, lacking prior echocardiographic expertise, exhibited significant improvement in both the efficiency and quality of the TEE examination.

## Clinical trial number

Not applicable.

## Supplementary Information

Supplementary Material 1.

## Data Availability

The data supporting the findings of this study are available from the corresponding author upon reasonable request. However, certain portions of the data are restricted due to proprietary interests related to the validation of the simulator developed by Medical Simulation Technologies. Access to these specific data will be limited to ensure the protection of the company's intellectual property.
