# Supplementary material for: Simulation training significantly improves the performance of novice students achieving the standard transesophageal echocardiography views
Source: BMC Med Educ. 2025 Mar 7;25:350. doi: 10.1186/s12909-025-06917-1 (PMC11887252; doi:10.1186/s12909-025-06917-1)
Supplement: Supplementary file 1 — Supplementary Material 1. [file 12909_2025_6917_MOESM1_ESM.docx]

**Table S1. Summary of the results between test 1 and test 2.**

|  | **Test 1** | **Test 2** | **P-value** |
| --- | --- | --- | --- |
| Number of students who passed the test | 5 | 13 | 0.039 |
| Median percentage of successfully achieved TEE views | 90.6% | 94% | 0.005 |
| Precision throughout the simulated TEE examination | 0.43±0.043 | 0.48±0.07 | <0.001 |
| Precision in ME Mitral Commissural view | 0.36 [IQR: 0.31-0.45] | 0.48 [IQR: 0.38-0.53] | 0.01 |
| Precision in ME Bicaval view | 0.40±0.12 | 0.49±0.15 | 0.02 |
| Precision in ME Asc Ao Sax view | 0.45±0.12 | 0.46±0.15 | 0.80 |
| Precision in TG Sax view | 0.25±0.11 | 0.27±0.13 | 0.43 |
| Total time to complete the simulated TEE examination | 968 s (IQR: 580-1151 s) | 390 s (IQR: 327-571 s) | <0.001 |
| Time of completion of ME Mitral Commissural view | 39 s (IQR:22-97 s) | 17 s (IQR: 12-25 s) | 0.002 |
| Time of completion of ME Asc Ao SAX view | 24 s (IQR: 18-47 s) | 17 s (IQR: 12-25 s); | 0.03 |
| Time of completion of ME Bicaval view | 38 s (IQR: 24-154 s) | 15 s (IQR: 10-38 s) | <0.001 |
| Time of completion of TG Basal view | 72 s (IQR: 37-108 s) | 31 s (IQR: 19-6 s) | <0.001 |
| RAR index (probe rotations) | 45236 (IQR: 32160-89143) | 31035 (IQR: 20552-61536) | 0.17 |
| RAR index (Ante/Retroflexation) | 16830 (IQR: 13574-30765) | 10230 (IQR: 5673-18603) | 0.005 |
| RAR index (probe rotations) for the ME Mitral Commissural view | 1177 (IQR: 601-4652) | 525.5 (IQR: 250.8-1972) | 0.001 |
| RAR index (Ante/Retroflexation) for the ME Mitral Commissural view | 695 (IQR: 518-2313) | 445 (IQR: 0-761) | 0.02 |
| RAR index (probe rotations) for the ME Asc Ao SAX view | 990.5 (IQR:314-1640) | 697 (IQR: 288.5-1217) | 0.007 |
| RAR index (Ante/Retroflexation) for the ME Asc Ao SAX view | 260 (IQR: 0-600) | 210 (IQR: 0-630) | 0.79 |
| RAR index (probe rotations) for the ME Bicaval view | 1892 (IQR: 1313-5749) | 1586 (694-3591) | <0.001 |
| RAR index (Ante/Retroflexation) for the ME Bicaval view | 680 (IQR: 0-3173) | 60 (0-653) | 0.004 |
| RAR index (probe rotations) for the TG Basal SAX view | 3225 (IQR: 2066-7604) | 2152 (IQR: 1412-4816) | <0.001 |
| RAR index (Ante/Retroflexation) for the TG Basal SAX view | 2125 (IQR: 913-4558) | 940 (IQR: 390-1760) | 0.02 |

**Abbreviations**: Ao- aorta, Asc – ascending, ME- mid-esophageal, RAR index - rapidity and randomity of the probe movement SAX- short- axis, TEE- transesophageal echocardiography, TG- transgastric
